# External Pelvic Measurement

**Published:** 1885-04

**Authors:** 


### An Easy Method of External Pelvic Measurement.

At the meeting of the New York Obstetrical Society, October 7th, 1884, (*The New York Medical Journal*, February 7th, 1885), Dr. Murray reported a case of Cæsarean section.

"When Dr. Murray was called, the patient was almost moribund, the child's head was fixed, and the position was such that the diameters of the pelvis could not be taken." "The point to which Dr. Murray wished to call attention was the difficulty, under the circumstances, of making out the diameter of the superior strait."

Professor Alex. J. C. Skene, M. D., reports "A Successful Case of Laparo-elytrotomy," in the *Annals of Surgery*, January, 1885. " On examination, the doctor found a vertex presentation of a fully developed child, while the antero-posterior diameter of the superior strait was estimated by him at less than two inches."

"As it was clearly evident that the delivery of a living child through the natural passages was an impossibility, and as we both further believed the mother's chances would be better if subjected to laparo-elytrotomy than if craniotomy were to be attempted, we advised the abdominal section."

In Dr. Murray's case, apparently no attempt was made to measure the pelvis. In Dr. Skene's case, "the doctor" found "the antero-posterior diameter of the superior strait was less than two inches."

In both cases, the indications for operation are clearly left indeterminate, so far as the general reader is concerned.

All who are familiar with Dr. Murray's sagacity, and Professor Skene's sound judgment, will be inclined to believe that the operation was indicated in each case. But such belief will rest upon the confidence reposed in the two surgeons, and not on positive evidence. Medicine, however, is not a matter of sentiment.

The two cases, to which allusion has just been made, have absolutely no scientific value, as bearing upon the indications for the operation, and a grave injustice is inflicted upon the reading medical world.

Apart from the natural regret that so much valuable material and labor is practically lost, the omission of accurate pelvic measurement excites wonder and surprise, at a time when approximately exact methods exist, and can be found in the modern text-books on midwifery.

The laborious investigations of Baudelocque, Litzmann, Michaelis, Dohrn, Nägele, Kiwisch, and a host of others, are totally disregarded.

Nor is it at all essential to measure by internal digital methods the antero-posterior diameter of the inlet.

By means of the sculptor's caliper-compasses of Baudelocque,

(1784), it is possible to determine the distance between the anterior-superior iliac spines, iliac crests, great trochanters, spine of last lumbar vertebra and the symphysis, or the *conjugata externa* s. *Baudelocqui.* From such data, it is possible to estimate with approximate accuracy the dimensions of the large and small pelvis.

We desire to call attention particularly to the simple and ingenious method of external pelvic measurement, suggested by Kiwisch in 1851.

The external circumference of the pelvis in living women, measured by means of a tape, applied to the extremity of the spine of the last lumbar vertebra, carried midway between the iliac crest and the great trochanter to the symphysis, and back to the starting point, has an average of ninety ctm. The development of the *panniculus adiposus* and the slender character of the bones exercise little influence upon this circumference. For every diminution of five ctm. in this circumference, it has been found that the antero-posterior diameter of the superior strait is diminished in length by one ctm.

Thus, an external pelvic circumference of 90 ctm. means a true conjugate of 11 ctm.; 85 ctm., 10 ctm.; 80 ctm., 9 ctm.; 75 ctm., 8 ctm.; 70 ctm., 7 ctm.; 65 ctm., 6 ctm.

Coxalgia and luxations of the hip joint, invalidate the method, for obvious reasons. None of these pathological conditions existed in the cases of Dr. Murray and Professor Skene.

Kiwisch's method has been subjected to continual application, since its introduction, in all the larger lying-in hospitals of Continental Europe.

Reliance is placed upon it particularly by Professor Carl Braun, of Vienna. It will be remembered that Professor Carl Braun has performed more Cæsarean sections than any living obstetrical surgeon. The number of Cæsarean sections in Vienna may be explained by the fabulous number of women, confined in the *Allgemeines Krankenhaus*, and the social condition of the peasant class.

